# Pro-Nerve Growth Factor Induces Activation of RhoA Kinase and Neuronal Cell Death

**DOI:** 10.3390/brainsci9080204

**Published:** 2019-08-19

**Authors:** Marina Sycheva, Jake Sustarich, Yuxian Zhang, Vaithinathan Selvaraju, Thangiah Geetha, Marla Gearing, Jeganathan Ramesh Babu

**Affiliations:** 1 Department of Nutrition, Dietetics, and Hospitality Management, Auburn University, Auburn, AL 36849, USA; 2 Center for Neuroscience Initiative, Auburn University, Auburn, AL 36849, USA; 3 Boshell Metabolic Diseases and Diabetes Program, Auburn University, Auburn, AL 36849, USA; 4 Department of Pathology and Laboratory Medicine, Emory University School of Medicine, Atlanta, GA 30322, USA

**Keywords:** NGF, proNGF, p75^NTR^, Alzheimer’s disease, RhoA kinase, neuronal death

## Abstract

We have previously shown that the expression of pro-nerve growth factor (proNGF) was significantly increased, nerve growth factor (NGF) level was decreased, and the expression of p75^NTR^ was enhanced in Alzheimer’s disease (AD) hippocampal samples. NGF regulates cell survival and differentiation by binding TrkA and p75^NTR^ receptors. ProNGF is the precursor form of NGF, binds to p75^NTR^, and induces cell apoptosis. The objective of this study is to determine whether the increased p75^NTR^ expression in AD is due to the accumulation of proNGF and Rho kinase activation. PC12 cells were stimulated with either proNGF or NGF. Pull-down assay was carried out to determine the RhoA kinase activity. We found the expression of p75^NTR^ was enhanced by proNGF compared to NGF. The proNGF stimulation also increased the RhoA kinase activity leading to apoptosis. The expression of active RhoA kinase was found to be increased in human AD hippocampus compared to control. The addition of RhoA kinase inhibitor Y27632 not only blocked the RhoA kinase activity but also reduced the expression of p75^NTR^ receptor and inhibited the activation of JNK and MAPK induced by proNGF. This suggests that overexpression of proNGF in AD enhances p75^NTR^ expression and activation of RhoA, leading to neuronal cell death.

## 1. Introduction

Alzheimer’s disease (AD) is a progressive neurodegenerative disease and is the primary cause of dementia in elderly individuals. The p75 neurotrophin receptor (p75^NTR^) plays several roles in AD pathogenesis, such as neuronal death [1,2], neuritic dystrophy [3], amyloid-beta generation [4], and Tau hyperphosphorylation [5,6]. p75^NTR^ expression level is found to be increased after neural injury and its signaling leads to neuronal death and dysfunction [7]. Studies from our laboratory [8] and Chakravarthy et al. [9] have shown that the expression of p75^NTR^ was enhanced in AD hippocampal tissues compared to control.

Nerve growth factor (NGF) mediates neuronal survival, differentiation, and maintenance of neurons [10], by binding to TrkA and p75^NTR^ receptors [11,12,13]. Pro-nerve growth factor (proNGF) is cleaved into NGF by matrix metalloproteinase (MMP) [14]. In our previous study, we showed that the expression of MMP-7 was reduced in AD hippocampus, which led to decreased levels of NGF and increased expression of proNGF [8]. ProNGF can promote neuronal apoptosis by binding to p75^NTR^ in neurodegenerative disease [15,16,17].

RhoA is activated by the overexpressed p75^NTR^, which in turn leads to neuronal death [18,19]. RhoA is a member of the Ras superfamily of GTPases and involved in neuronal development, survival, and death [20]. RhoA switches between the GDP-bound inactive and active forms [20]. Activation of RhoA induces neuronal death [18,21] and inactivation of RhoA leads to neuronal differentiation [22].

Previous studies from our lab have shown that in AD human hippocampal samples, the expression of proNGF and p75^NTR^ was increased compared to control [8]. In this study, we demonstrate that in AD, the increase in proNGF induces neuronal cell death through p75^NTR^ and Rho activation. Inhibition of RhoA kinase reduced the expression of p75^NTR^ and inhibited the proNGF induced neuronal death by abrogating the activation of JNK and p38MAPK.

## 2. Materials and Methods

### 2.1. Reagents and Antibodies

Anti-p75^NTR^ (Cat. # G323A; 1:1000 dilution) was purchased from Promega (Madison, WI, USA). Anti-RhoA (Cat. # 05-778; 1:1000 dilution) and agarose conjugated rhotekin- Rho-binding domain (RBD) was purchased from Millipore (Billerica, MA, USA). Phospho-p38MAPK (Cat. # 9221; 1:1000 dilution) and non-phospho p38MAPK antibodies (Cat. # 9212; 1:1000 dilution), JNK (Cat. # 9252S; 1:1000 dilution) and phospho JNK (Cat. # 9251S; 1:1000 dilution), cleaved caspase-3 antibodies (Cat. # 9664; 1:1000 dilution) were purchased from Cell signaling (Danvers, MA, USA). Cleaved poly ADP-ribose polymerase (PARP) antibody (Cat. # 556494; 1:1000 dilution) was obtained from BD Bioscience Pharmingen (San Diego, CA, USA). ProNGF was obtained from Alomon (Israel), NGF (Cat. # 04-1142) from Bioproducts for science (Indianapolis, IN, USA), and Y-27632 (Cat. # 10005583) was purchased from Cayman Chemical Company (Ann Arbor, MI, USA). Enhanced chemiluminescence (Cat. # 32106) was from Thermo Scientific (Waltham, MA, USA), MTT Cell Proliferation Assay Kit (Cat. # ab211091) was procured from abcam (Cambridge, MA, USA), and all other reagents were obtained from Sigma-Aldrich.

### 2.2. Brain Tissue

The human brain tissues used in this study were obtained from Emory University Alzheimer’s Disease Center Brain Bank (Atlanta, GA, USA). Frozen samples of hippocampus from six AD cases aged 58–90 (mean = 69) and 6 control subjects aged 59–94 (mean = 70) were used for this study. The same brain tissues were used in Zheng et al. [8].

### 2.3. Cell Culture

PC12 rat pheochromocytoma cells were cultured in Dulbecco’s modified Eagle’s media (DMEM) supplemented with 10% heat-inactivated horse serum, 5% fetal bovine serum, and antibiotics (100 units/mL; streptomycin and penicillin) on 100 mm plates coated with 150 μL Type I collagen. Cells were incubated at 37 °C in a humidified atmosphere containing 5% CO_2_ and 95% air. Cell culture medium was changed once a week. The cells were treated with proNGF (50 ng/mL) or NGF (50 ng/mL) in starved media overnight at 37 °C before cell lysis. To study Y-27632 (ROCK inhibitor) effects on proNGF stimulation, cells were treated with proNGF (50 ng/mL) in the presence or absence of Y-27632 (1 μM) overnight at 37 °C before cell lysis. All the experiments were replicated three times.

### 2.4. Western Blot

At the end of treatments, cell were lysed with HEPES lysis buffer (50 mM HEPES [pH 7.6], 150 mM NaCl, 20 mM sodium pyrophosphate, 10 mM NaF, 20 mM beta-glycerophosphate, 1% Triton, 10 μg/mL leupeptin, 10 μg/mL aprotinin, 1 mM Na_3_VO_4_, and 1 mM PMSF). The protein concentrations were analyzed using the Bradford procedure (Bio-Rad, Hercules, CA, USA) using bovine serum albumin as a standard for all samples. The samples (40 µg of protein) were boiled in sodium dodecyl sulfate polyacrylamide gel electrophoresis (SDS-PAGE) sample buffer and resolved on SDS-PAGE gels, transferred onto polyvinylidene difluoride membrane, and analyzed by Western blotting with appropriate antibodies.

### 2.5. RhoA Kinase Activity

RhoA kinase activity was determined by pull-down assay. Brain homogenates or PC12 cell lysates were incubated with agarose-conjugated rhotekin RBD agarose beads for 45 min at 4 °C and washed three times with lysis buffer. The beads were boiled with SDS-PAGE sample buffer to release active RhoA. Bound RhoA was detected by Western blotting with the anti-RhoA antibody.

### 2.6. MTT Cell Viability Assay

MTT (3-(4,5-dimethylthiazol-2-yl)-2,5-diphenyltetrazolium bromide) assay was used in five replicates to measure the cell viability as per the manufacturer protocol. PC12 cells at 7500/well were seeded in Type I collagen-coated 96-well plate in DMEM containing 10% heat-inactivated horse serum, 5% fetal bovine serum, and antibiotics. The day after seeding, the cells were serum-starved and treated overnight with either pro-NGF (50 ng/mL), Y-27632 (1 µM), or both. Treatment media was replaced with 50 µL MTT reagent and 50 µL serum-free media and incubated at 37 °C for 3 h. After incubation, 150 µL of MTT solvent was added, wrapped with aluminum foil, and incubated in an orbital shaker for 15 min. Absorbance was read at 590 nm using Spectramax M2 plate reader (Molecular Devices, San Jose, CA, USA). Results were expressed as a percentage of the control group.

## 3. Results

**Figure 1:**
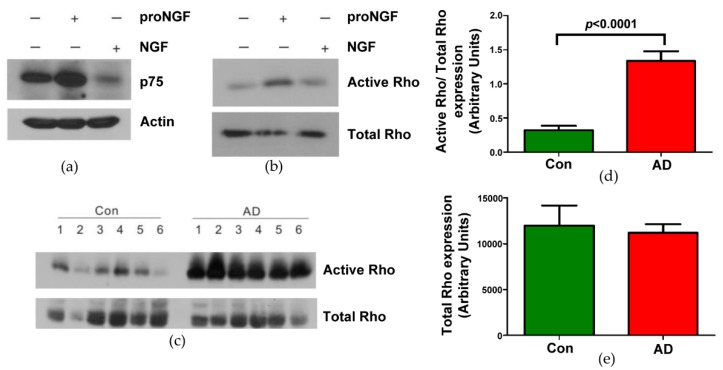
Pro-nerve growth factor (NGF) increased the expression of p75^NTR^ and activation of Rho. PC12 cells were treated with pro-NGF (50 ng/mL) or NGF (50 ng/mL) overnight. The cells were lysed and (**a**) Western blotted with anti-p75, anti-actin, (**b**) lysates were subjected to pull-down assay with agarose conjugated rhotekin- Rho-binding domain (RBD) followed by Western blot with Rho antibody. (**c**) Homogenates of postmortem age matched control and Alzheimer’s disease (AD) human hippocampal tissues were subjected to pull-down assay with agarose conjugated rhotekin-RBD to detect active Rho. (**d**) Quantification of the Western blot of active Rho shown in the top panel of (**c**). Control was compared to AD human hippocampal tissues (n = 6; *p* < 0.0001). (**e**) Bar graph quantifying the Western blot of total Rho shown in the bottom panel of (**c**). Expression shows no difference between control and AD patients.

ProNGF can induce neuronal apoptosis due to its high affinity to bind p75^NTR^ [23,24]. The effect of proNGF signaling is dependent upon the expression levels of p75^NTR^ [25,26]. In our previous studies, postmortem AD human hippocampal samples showed an increase in expression of proNGF and p75^NTR^ receptor compared to control samples [8]. Therefore, as the first step, we evaluated whether proNGF stimulation can increase the expression of p75^NTR^. PC12 cells were treated overnight with vehicle, proNGF, or NGF. The cells were lysed and Western blotted with p75^NTR^ and actin antibodies. Figure 1a shows that cells treated with proNGF upregulated the expression of p75^NTR^ compared to control and NGF treated cells. Actin levels were determined as a positive control to check equal loading of all the samples. P75^NTR^ can lead to RhoA activation and neuronal death [18,19,21]. Therefore, we examined the activation of the RhoA kinase pathway implicated in neuronal death. PC12 cells treated with vehicle, proNGF, or NGF were subjected to pull-down assay and Western blotted with Rho antibody. As shown in Figure 1b, activation of Rho kinase was increased in proNGF-stimulated cells compared to control and NGF. The expression of total Rho was equal in all the cell lysates. In addition to this, we also examined the Rho kinase activity in AD brain since proNGF and p75^NTR^ were increased in those tissues. We used postmortem hippocampal samples derived from six aged control subjects and six AD patients [8]. Rho kinase activity was measured by pull-down assay in the tissue homogenates. As shown in Figure 1c,d, Rho kinase was significantly activated in AD (*p* < 0.0001) compared to control brain hippocampus. There was no significant difference in the expression of total Rho between the control and AD brain homogenates as shown by Western blot (Figure 1c,e).

**Figure 2:**
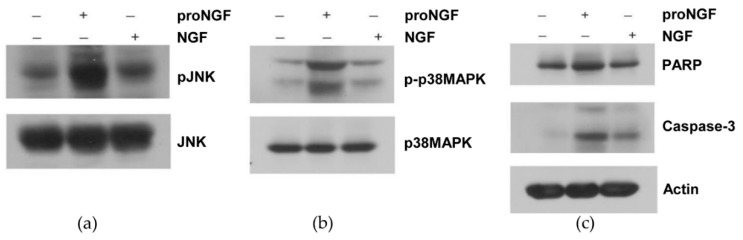
Pro-NGF induced activation of JNK, p38 MAPK pathway, and expression of apoptotic markers in PC12 cells. PC12 cells were treated with pro-NGF (50 ng/mL) or NGF (50 ng/mL) overnight. The cells were lysed and Western blotted with (**a**) phospho and non-phospho-JNK antibodies, (**b**) phospho and non-phospho-p38MAPK antibodies, and (**c**) poly ADP-ribose polymerase (PARP), caspase-3, actin antibodies.

JNK and p38MAPK signaling pathway are critical for the induction of neuronal apoptosis [27,28]. To examine the effect of proNGF on receptor signaling, the activation of JNK and p38MAPK was determined as downstream targets. PC12 cell lysates were stimulated overnight with vehicle, proNGF, or NGF. The cell lysates were Western blotted with JNK and p38MAPK or their specific-phospho antibodies. The results suggest that phosphorylation of JNK and p38MAPK was increased by proNGF over control or NGF-treated cells (Figure 2a,b). The same lysates were also analyzed for apoptotic markers such as cleaved PARP and caspase-3. The expression level of cleaved PARP and caspase-3 was increased in cells treated with proNGF compared with control or NGF-treated cells. (Figure 2c).

**Figure 3:**
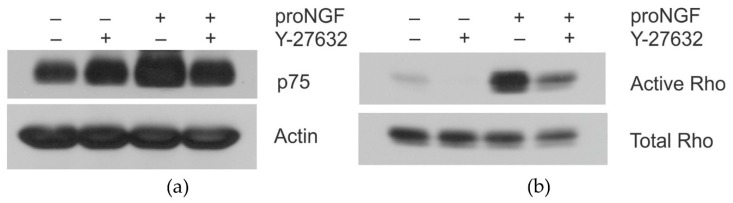
Inhibition of Rho activation reduced the expression of p75^NTR^ in PC12 cells. PC12 cells were treated overnight with either pro-NGF (50 ng/mL) or Rho kinase inhibitor, Y-27632 (1 µM), or both. The cells were lysed and (**a**) Western blotted with anti-p75^NTR^, anti-actin (**b**) pull-down assay with agarose conjugated rhotekin-RBD to detect the activation of Rho.

The expression of p75^NTR^ is increased in AD [8,9] and by overexpression of proNGF (Figure 1a). The increased expression of proNGF and p75NTR, in turn, activates RhoA kinase (Figure 1b,c). We further wanted to determine whether inhibiting Rho kinase activity would reduce the expression of p75^NTR^. We pretreated the PC12 cells overnight with pro-NGF, Rho kinase inhibitor, Y-27632, or both. The cell lysates were Western blotted with p75^NTR^ and actin antibodies (Figure 3a). The expression of p75^NTR^ was reduced by attenuating the Rho kinase activity. Activation of Rho kinase in the same lysates was also detected by pull-down assay. Figure 3b suggests that RhoA kinase activity was reduced by the addition of Y-27632. This clearly explains that Rho kinase activity causes an increase in the expression of p75^NTR^.

**Figure 4:**
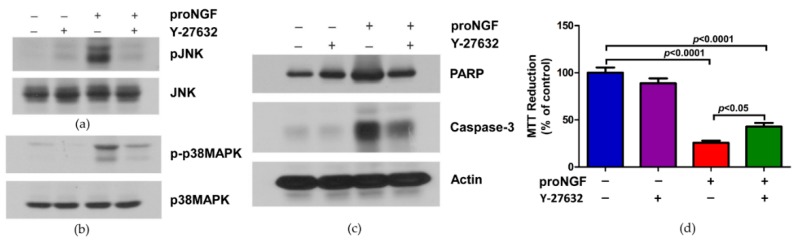
Inhibition of Rho activation reduced the activation of JNK, p38MAPK, and apoptotic markers expression. PC12 cells were treated overnight with either pro-NGF (50 ng/mL) or Rho kinase inhibitor, Y-27632 (1 µM), or both. The cells were lysed and Western blotted with (**a**) phospho and non-phospho-JNK antibodies, (**b**) phospho and non-phospho-p38MAPK antibodies, (**c**) PARP and caspase-3 antibodies, and (**d**) cell death was measured by MTT assay. The bar graph shows the mean and standard deviation (n = 5).

Next, we determined whether attenuating the Rho kinase activity will block the phosphorylation of JNK and p38MAPK. The cell lysates treated with either proNGF, Y-27632, or both were Western blotted with phospho and non-phospho antibodies of JNK and p38MAPK antibodies. The Rho kinase inhibitor Y-27632 reduced the activation of JNK and p38MAPK compared to proNGF alone treated cells (Figure 4a,b). The Rho kinase inhibitor, as shown in Figure 4c, also reduced the expression of cleaved-PARP and caspase-3. In addition to Western blot results, cell death was assessed by MTT assay. ProNGF and proNGF with inhibitor treated groups showed a significant increase in the cell death compared to control group. However, addition of the RhoA inhibitor along with the proNGF significantly increased (*p* < 0.05) the cell viability compared to proNGF alone treated cells. Taken together, these findings suggest that proNGF induces cell death through activation of Rho kinase.

## 4. Discussion

ProNGF, the nerve growth factor precursor protein expression level, is increased in Alzheimer’s disease brain and cerebrospinal fluid [8,29]. The balance between the expression levels of proNGF and NGF determines cell survival or cell death [30]. The expression of p75^NTR^ is also enhanced in AD hippocampal brain compared to control [8,9]. However, the relationship between the expression of proNGF and p75^NTR^ is still not completely understood. Our findings reveal that stimulation of PC12 cells with proNGF increases the expression of p75^NTR^ compared to NGF. P75^NTR^ participates in several signaling pathways to promote cell survival, apoptosis, differentiation, Schwann cell myelination, and sensory neuron development [31,32,33,34]. ProNGF is known to bind p75^NTR^ and promote neuronal apoptosis in neurodegenerative disease [15,16,17].

Interestingly, our results showed that Rho kinase was activated in postmortem human AD compared to control hippocampus. However, these results can also be further validated by imaging experiments. p75^NTR^ is found to activate RhoA and promote neuronal death [21,35]. We also found that proNGF treatment increases the activation of JNK/p38MAPK and cell death in PC12 cells. Activation of RhoA induces neuronal death by activating p38MAPK [18,19,21,35].

Inhibition of Rho kinase might provide a therapeutic target for several neurological disorders such as AD, stroke, spinal cord injury, inflammatory, and demyelinating diseases [22,36,37,38,39]. We used Y27632, [(R)-(+)-trans-N-(4-Pyridyl)-4-(1-aminoethyl)-cyclohexanecarboxamide·2HCl], as Rho kinase inhibitor [40]. In this study, we found that this Rho kinase inhibitor reduced the expression of p75^NTR^, which was increased by proNGF. Activation of downstream signaling of JNK and p38MAPK was also inhibited by Y27632 and attenuated the cell death induced by proNGF in PC12 cells. This result was also supported by the MTT assay, which showed a significant increase in cell viability by the treatment of Rho kinase inhibitor along with proNGF compared to proNGF only treated cells. These findings suggest that blocking activation of Rho kinase can reverse proNGF-induced cell death through p75^NTR^ in Alzheimer’s disease.

The expression of p75^NTR^ and RhoA kinase activity was increased by proNGF compared to NGF in PC12 cells. The expression of active RhoA kinase was also increased in human AD hippocampus. Inhibition of RhoA kinase reduced the expression of p75^NTR^ receptor and also inhibited the neuronal death by abrogating the activation of JNK and p38MAPK induced by proNGF.
